# *Drosophila* mutants lacking the glial neurotransmitter-modifying enzyme Ebony exhibit low neurotransmitter levels and altered behavior

**DOI:** 10.1038/s41598-023-36558-7

**Published:** 2023-06-27

**Authors:** Meghan Pantalia, Zhi Lin, Samantha J. Tener, Bing Qiao, Grace Tang, Matthew Ulgherait, Reed O’Connor, Rebecca Delventhal, Julia Volpi, Sheyum Syed, Nissim Itzhak, Julie C. Canman, María Paz Fernández, Mimi Shirasu-Hiza

**Affiliations:** 1 Department of Genetics and Development, Columbia University Irving Medical Center, New York, NY 10032 USA; 2 Department of Physics, University of Miami, Coral Gables, FL 33146 USA; 3 Department of Neuroscience and Behavior, Barnard College, New York, NY 10027 USA; 4 Department of Biology, Lake Forest College, Lake Forest, IL 60045 USA; 5 Division of Human Genetics and Metabolic Disease, Children’s Hospital of Philadelphia, Philadelphia, PA 19104 USA; 6 Department of Pediatrics, Biochemistry and Biophysics, Perelman School of Medicine, University of Pennsylvania, Philadelphia, PA 19104 USA; 7 Department of Pathology and Cell Biology, Columbia University Irving Medical Center, New York, NY 10032 USA

**Keywords:** Behavioural genetics, Sleep, Social behaviour

## Abstract

Inhibitors of enzymes that inactivate amine neurotransmitters (dopamine, serotonin), such as catechol-O-methyltransferase (COMT) and monoamine oxidase (MAO), are thought to increase neurotransmitter levels and are widely used to treat Parkinson's disease and psychiatric disorders, yet the role of these enzymes in regulating behavior remains unclear. Here, we investigated the genetic loss of a similar enzyme in the model organism *Drosophila melanogaster*. Because the enzyme Ebony modifies and inactivates amine neurotransmitters, its loss is assumed to increase neurotransmitter levels, increasing behaviors such as aggression and courtship and decreasing sleep. Indeed, *ebony* mutants have been described since 1960 as "aggressive mutants," though this behavior has not been quantified. Using automated machine learning-based analyses, we quantitatively confirmed that *ebony* mutants exhibited increased aggressive behaviors such as boxing but also decreased courtship behaviors and increased sleep. Through tissue-specific knockdown, we found that *ebony*’s role in these behaviors was specific to glia. Unexpectedly, direct measurement of amine neurotransmitters in *ebony* brains revealed that their levels were not increased but reduced. Thus, increased aggression is the anomalous behavior for this neurotransmitter profile. We further found that *ebony* mutants exhibited increased aggression only when fighting each other, not when fighting wild-type controls. Moreover, fights between *ebony* mutants were less likely to end with a clear winner than fights between controls or fights between *ebony* mutants and controls. In *ebony* vs. control fights, *ebony* mutants were more likely to win. Together, these results suggest that *ebony* mutants exhibit prolonged aggressive behavior only in a specific context, with an equally dominant opponent.

The *ebony* gene encodes an enzyme, a β-alanyl biogenic amine synthase, that catalyzes the addition of β-alanine to biogenic amines (histamine, dopamine, serotonin, and octopamine), which inhibits these neurotransmitters and prepares them for recycling^1,2^. In a 1960 study of mating behavior, M.E. Jacobs observed that *ebony* mutants fight more than “light flies”^3^. Though this manuscript is typically cited as the first genetic study of aggressive behavior in *Drosophila*^4^, the aggressive behavior of *ebony* mutants has not been characterized or quantified. M.E. Jacobs went on to note that *ebony* mutants also exhibit less courtship behavior^3^. These two results appear contradictory because courtship and aggression are both positively regulated by biogenic amine neurotransmitters; changes in courtship and aggression are typically correlated, with increases in one behavior normally associated with increases in the other^5–9^. Since Jacob’s work, no one has characterized *ebony* aggression; Suh and Jackson^10^ showed that *ebony* activity impacts circadian rhythms, possibly via its role in neurotransmitter regulation, but did not examine courtship or aggression^10^. Here, we quantitatively examined *ebony* mutants to definitively test the role of this enzyme in regulating three different behaviors affected by biogenic amines: aggression, courtship, and sleep.

Widely used as a phenotypic marker because *ebony* mutants have very dark pigmentation, *Drosophila ebony* is expressed in the epidermal epithelium, the oenocytes (abdominal secretory cells), and in several types of glial cells. Importantly, *ebony* is not expressed in neurons^1,11–13^. Glial cells have long been known to perform essential tasks in the brain, including the provision of metabolic and structural support to neurons^14–16^. Recent research has led to increased appreciation for a more complex role of glial cells in neuronal functions, including the regulation of behavior—in part due to the role of glia in the synthesis, uptake, and regulation of neurotransmitters^2,17–19^. The extent to which glial cells modulate behavior through their regulation of neurotransmission remains understudied; there have only been limited studies directly examining the role of glia-mediated regulation of neurotransmission in complex behaviors (e.g., courtship and aggression)^13,18,20^. Given the genetic tractability and established behavioral assays in *Drosophila*, this model is well-equipped to advance our understanding of how glial regulation of neurotransmission affects diverse behaviors.

While there is no mammalian homolog of *ebony*, several genes might act as functional analogs: mammalian glutamine synthetase, which inactivates the biogenic amine glutamate by converting to glutamine in astrocytes, thereby reducing glutamate levels in the synapse^20^; catechol-O-methyltransferase (COMT), which inactivates catecholamine neurotransmitters (dopamine, epinephrine, and norepinephrine) by introducing a methyl group^21^; and monoamine oxidases (MAOs), which inactivate monoamines such as serotonin, dopamine, and tyramine by catalyzing their oxidative deamination^22^. As stated above, because biogenic amines positively regulate behaviors such as sex drive and aggression^23,24^, inhibition of enzymes that inactivate these neurotransmitters are predicted to increase these behaviors–for example, increasing aggression, increasing sex drive, and decreasing sleep.

In this work, we used *ebony*^*11*^ mutants to study how the loss of this biogenic amine-inactivating enzyme impacts these behaviors. We used machine learning-based algorithms to quantitatively analyze specific courtship and aggression behaviors (FlyTracker^25^ and JAABA^26^; 95.6–100% accuracy with ground truthing). Consistent with previous observations^3,27^, we found that *ebony* mutants exhibited decreased courtship behaviors and increased aggression; we also observed increased sleep. Through tissue-specific knockdown of *ebony* with RNAi, we showed that these behavioral phenotypes can be attributed to its role specifically in glial cells. To understand the basis for these behaviors, we quantified levels of biogenic amines in *ebony*^***11***^ mutant brains and found that they contained significantly decreased levels of histamine, dopamine, and serotonin relative to control brains, with no difference in octopamine levels. This result is consistent with decreased courtship and increased sleep but not with increased aggression. When we examined fights between *ebony* mutants and controls, we found that *ebony* mutants were not inherently aggressive; their prolonged fighting appeared to be due to an inability to establish dominance with an equally dominant opponent. Taken together, these findings provide the first quantitative characterization of the role of this glial enzymatic modifier of biogenic amines in regulating behavior, particularly aggression.

## Results

### *ebony* mutants lack Ebony protein

**Figure 1:**
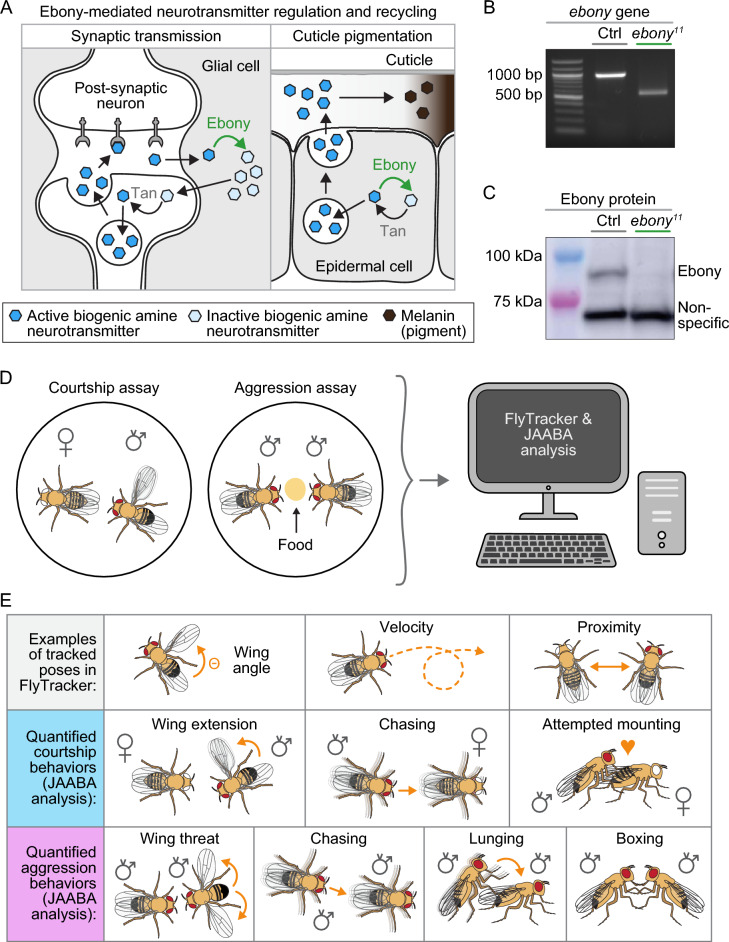
Validation of *ebony*^*11*^ mutant and quantitative analysis pipeline for *Drosophila* courtship and aggression behaviors. (**A**) Schematic showing the role of Ebony in recycling biogenic amines via inactivation of neurotransmitters in glial and epidermal cells. (**B**) PCR of *ebony* mutants and controls, revealing that *e*^*11*^ mutants have the expected ~500bp deletion. (**C**) western blot showing that *ebony*^*11*^ mutants lack Ebony protein. (**D**) schematic of aggression and courtship assays, which were video-recorded and analyzed by FlyTracker and JAABA machine learning classifiers and (**E**) schematic depicting fly behavioral parameters that were annotated by FlyTracker and used by JAABA machine learning classifiers to quantify specific courtship and aggression behaviors.

Previous research supports a model in which Ebony protein modifies and inactivates neurotransmitters taken up by glia from the synaptic cleft^2^. These modified, inactivated neurotransmitters are then transported back to pre-synaptic neurons, where they are reactivated by Tan, a hydrolase which removes the β-alanine group from the neurotransmitter for its re-use in synaptic transmission (Fig. 1A). To explore the role of *ebony* (*e*) in behavior, we selected the *e*^*11*^ allele, which we validated for loss of Ebony protein (see our brief discussion of the more commonly used but unvalidated *e*^*1*^ allele in Discussion and Methods). *e*^*11*^ mutants have the dark cuticle color characteristic of all *ebony* mutants; *ebony* is expressed in both glia and epidermal cells and loss of Ebony causes accumulation of biogenic amines in epidermal cells, which darkens the cuticle (Figs. 1A, S1A). PCR analysis confirmed that this *e*^*11*^ allele contained the predicted genomic deletion reported by Rossi et al.^28^. Primers around this expected deletion site amplified a DNA fragment that was ~ 500 bp smaller in *e*^*11*^ mutants than controls (Fig. 1B). While *e*^*11*^ mutants exhibited increased, likely compensatory, *ebony* mRNA expression relative to controls by qRT-PCR (Fig. S1B), we confirmed by western blot analysis that *e*^*11*^ mutants lacked Ebony protein (Fig. 1C; whole blot in Fig. S1C). Thus, our data confirm the mutation and loss of Ebony protein in *e*^*11*^ mutants (hereafter referred to as *ebony* mutants).

### Automated quantification of *Drosophila* courtship and aggression with tracking and machine learning software

Because biogenic amine neurotransmitters are known to influence courtship and aggression^7,29–34^, we tested if our confirmed *ebony* mutant males exhibited changes in their courtship or aggressive behaviors. To measure courtship and aggression, we used an automated machine-learning based method combining the Caltech FlyTracker^25^ and the Janelia Automatic Animal Behavior Annotator (JAABA), established by the Branson lab at HHMI Janelia Farm^26^ (Fig. 1D). We filmed 10-min videos of flies engaged in either courtship or aggression assays and used the Caltech FlyTracker to obtain information about each fly’s position, orientation relative to another fly, poses, and trajectories (Fig. 1E). A machine learning classifier is an algorithm to automatically categorize data within that “class”; for this work, the “class” is a behavior such as chasing or setting wings at a specific angle. We used a subset of annotated videos (training data) to train the JAABA machine learning classifiers to detect the specific, annotated behaviors, validated the trained JAABA classifiers with a second subset of manually-scored control videos, and then used the JAABA classifiers to quantify courtship and aggression behaviors in experiments.

To quantify courtship behaviors, we trained JAABA to detect wing extension, chasing, and mounting without copulation, three parameters of courtship (Fig. 1E). We used ground truthing strategy to validate the accuracy of our classifiers for courtship analysis (Fig. S1D). Ground truthing uses all the behavior video frames that have been annotated by the user to make a classifier (training data), and the classifier then quantifies those behaviors for videos it has never seen before (testing data). The user manually annotates the novel “testing” videos, and the computer outputs the accuracy of the classifier’s predictions in comparison. We made a separate classifier to quantify chasing behavior in courtship, as male chasing of females in courtship may have subtle differences in speed and orientation from male chasing of other males in aggression. For courtship, the classifiers agreed with the user for 99.2–100% of the testing data frames. To quantify aggressive male behaviors, we trained classifiers to detect wing threat, chasing, lunging, and boxing (Fig. 1E) and performed the same ground truthing analysis (Fig. S1D). For aggression, these classifiers agreed with the user for 95.6–100% of the testing data frames. Thus, our classifiers for both aggression and courtship were highly accurate and precise, facilitating video analysis at higher throughput than manual analysis.

### *ebony* mutants display decreased courtship behavior towards females

**Figure 2:**
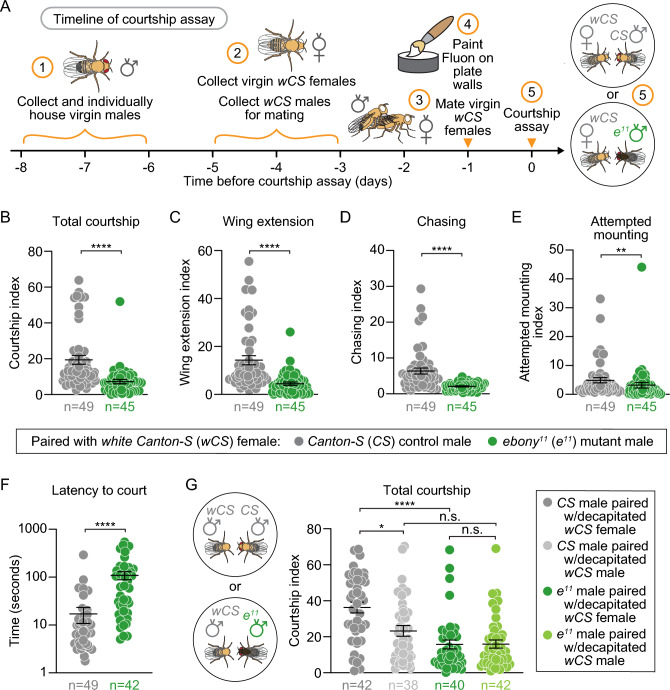
*ebony* mutant males court females, but not males, less than controls. (**A**) Schematic of our courtship assay with mated *wCS* females. (**B–F**) When courting mated *wCS* females, *ebony* mutants (green), compared to controls (gray), exhibited: (**B**) decreased total courtship (p < 0.0001); (**C**) decreased wingsong behavior (p < 0.0001); (**D**) decreased chasing behavior (p < 0.0001); (**E**) decreased attempted mounting (p = 0.0082); and (**F**) increased latency to first courting event (p < 0.0001). (**G**) In courtship assays with decapitated wCS virgin females or males, control males courted females (dark gray) more than males (light gray) (p = 0.0211) and more than *ebony* males courted females (dark green) (p < 0.0001). There was no difference in courtship levels between control males (light gray) and *ebony* males (light green) with decapitated males (p = 0.2585) or between *ebony* males with females (dark green; p>0.9999). Each data point represents a single male (n=number of flies); p-values were obtained by Mann-Whitney U test (**B–F**) and Kruskal-Wallis test with Dunn’s post hoc test (**G**). Averages are shown with error bars representing SEM. (See Table S1 for n and statistical analysis for each experiment; n.s.= p > 0.05, * = p < 0.05, **= p < 0.01, **** = p < 0.0001).

To assay changes in courtship behavior for *ebony* mutants, we video-recorded the interactions between pairs of 6–8 day-old virgin males and 3–5 day-old *wCS* (*w*^*-*^* Canton S* strain) females in a 16 mm bottom-lit chamber for 10 min (Fig. 2A). For each male, we determined the courtship index (CI), a standard metric in the field which reflects the percentage of video frames in which the male exhibited any of several types of courtship behavior. Unless otherwise noted, females were mated to prevent copulation and to ensure a window of courtship behavior extending for the duration of video recording. Using our automated analysis programs, we found that *ebony* mutant males spent significantly less time exhibiting courtship behavior relative to controls (Fig. 2B). In assessing individual courtship behaviors, we found that *ebony* mutants spent significantly less time on wing extension (Fig. 2C), chasing (Fig. 2D), and attempted mounting (Fig. 2E) behavior than controls. We also measured the latency to court, or the time elapsed from the start of the recording to the first courtship behavior; *ebony* males exhibited an average courtship latency of 109 s while *CS* males had an average courtship latency of 17 s (Fig. 2F). Thus, *ebony* males exhibited reduced courtship index and were slower to initiate their first courtship attempt compared to *CS* control males.

To confirm that this phenotype is not specific to mated females, we quantified the CI of *ebony* males with virgin females (Fig. S2A). Virgin females have different cuticular hydrocarbon profiles than mated females, which are known to influence male courtship behavior^35,36^. Consistent with our results with mated females, *ebony* mutant males displayed a reduction in total courtship with virgin females (Fig. S2B). *ebony* males had an average courtship latency of 138 s, while *CS* males waited, on average, 47 s before initiating courtship (Fig. S2C). We also measured the percentage of pairs with successful copulation within the 10-min observation period. When paired with a virgin female, only 7% of the *ebony* mutant males achieved copulation within 10 min compared to 67% of the control males (Fig. S2D). Thus, *ebony* males display defects in courtship toward both mated and virgin females and copulate with virgin females less successfully than control males.

To test if this decrease in courtship behavior was specific to male–female interactions, we tested whether male-male courtship was also affected in *ebony* mutants. Male-male courtship can be induced by biogenic amine neurotransmitters and substrates of Ebony, such as dopamine^30^. For this assay, we decapitated both virgin male and female targets to prevent aggression from male targets toward the male being tested. Consistent with our results above, *ebony* males courted decapitated virgin females significantly less than *CS* males; in contrast, *ebony* males courted decapitated virgin males at the same rate as controls (Fig. 2G). Interestingly, while controls showed a courtship preference for females, there was no difference in courtship indices for *ebony* males with decapitated virgin females or males. Thus, *ebony* mutants exhibit decreases in male–female courtship relative to controls, with no difference in male-male courtship and no preference for courting either sex.

### *ebony* mutants display increased boxing and wing threat behaviors

**Figure 3:**
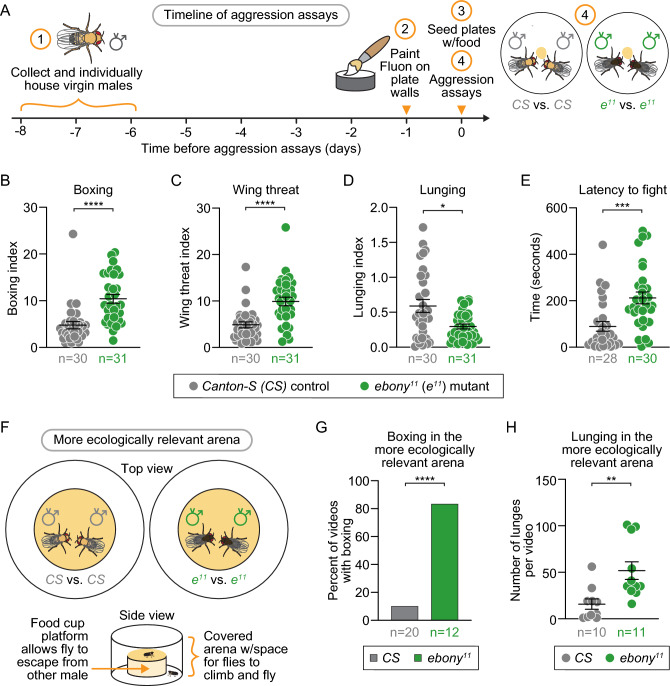
*ebony* mutants spend more time exhibiting “hyper-aggressive” behaviors and show a greater latency to initiate fighting. (**A**) Schematic of aggression assay. (**B–E**) In fights between *ebony* mutants (green), compared to fights between controls (gray), *ebony* mutants exhibited: (**B**) increased boxing behavior (p<0.0001); (**C**) increased wing threat behavior (p<0.0001); (**D**) decreased lunging behavior (p=0.0497); and (**E**) greater latency to first fighting event (p=0.0002); each data point represents a video of two flies (n=number of videos). (**F–H**) In aggression assays using a more ecologically relevant arena (**F**) with *ebony* mutants fighting each other (green), compared to controls fighting each other (gray), *ebony* mutants had: (**G**) a greater percentage of videos with boxing behavior (p<0.0001, n=number of videos); and (**H**) increased numbers of lunges per video (p=0.0025; n=number of flies). p-values were obtained by Mann-Whitney U test (**B–E**, **H**) and Fisher’s exact test (**G**); averages are shown with error bars representing SEM. (See Table S1 for n and statistical analysis for each experiment; n.s.= p>0.05, *=p<0.05, ** = p<0.01, *** = p<0.001, **** = p<0.0001.)

While it is widely accepted that *ebony* mutants are "hyper-aggressive", this characterization appears to be mainly based on two qualitative observations^3,27^. To quantitatively test this, we video-recorded the interactions between pairs of 6 to 8-day-old virgin males in a 16 mm bottom-lit chamber for 10 min and used the automated analysis described above to measure aggressive behavior. Each chamber contained a small area of food as a territory/resource to induce aggressive behavior (Fig. 3A). To quantify aggressive behaviors, we measured behavioral indices similar to courtship behavioral indices, reflecting the percentage of frames or time spent engaging in this behavior (Fig. 3B-D). We observed that *ebony* mutants spent dramatically more time engaged in boxing than controls (Fig. 3B); boxing is considered one of the most aggressive behaviors of flies^37^. We also determined the total number of videos in which we observed boxing; *ebony* mutants exhibited boxing in 90.3% of their videos, compared to 63.3% for *CS* controls (Fig. S3A). *ebony* mutants also displayed increased wing threat behavior relative to controls (Fig. 3C). Unexpectedly, relative to controls, *ebony* mutants exhibited decreased chasing behavior (Fig. S3B) (not considered a highly aggressive behavior as chasing is also seen in courtship) and decreased lunging (Fig. 3D), though boxing behavior can be considered reciprocal lunging. We also found that *ebony* mutants had an increased latency for aggressive behaviors relative to controls, similar to the latency in courtship behavior. That is, *ebony* males were slower to start fighting with each other (latency average of 212 s), than did controls, who fought with each other after an average of 90 s (Fig. 3E). Thus, *ebony* males exhibited an increase in specific aggressive behaviors such as boxing and wing threat.

Because the small, flat arena required for our automated analysis could itself increase aggressive behavior^38^, we confirmed these results in an assay with a more ecologically relevant arena with lower throughput analysis. We used a larger chamber with a raised food cup, as described by Fernandez et al.^39^ (Fig. 3F). Consistent with our results above, in the more ecologically relevant arena, significantly more videos of *ebony* mutants exhibited boxing relative to videos of controls (Fig. 3G) and *ebony* mutants exhibited more lunging behavior than controls (Fig. 3H). Lunging is a more commonly observed aggressive behavior than boxing, also exhibited specifically by males^37^. Latency to lunge, while trending to a shorter time for *ebony* mutants, was not significantly different from controls (Fig. S3C). Thus, our results suggest that, regardless of arena, *ebony* males exhibit increased aggression.

### *ebony* mutants exhibit increased sleep duration and sleep consolidation

**Figure 4:**
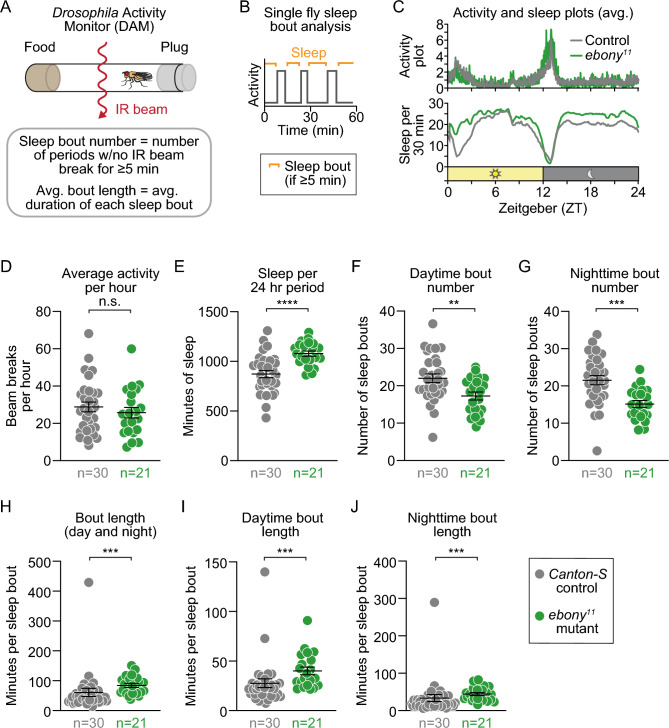
*ebony* mutants have fewer but longer sleep bouts and similar activity levels compared to controls. (**A–B**) Schematics of: (**A**) the setup of a Drosophila Activity Monitor (DAM); and (**B**) typical bouts of activity (wake) and inactivity (sleep) over a 1-hour period. (**C**) Plots show the average activity (top) and sleep duration per 30 minutes (bottom) over a 24-hour period for control flies (gray) and *ebony* mutants (green). (**D–J**) Relative to controls (gray), *ebony* mutants (green) exhibited: (**D**) similar average activity per hour (p = 0.4372); (**E**) increased sleep amount over a 24-hour period (p < 0.0001); (**F**) decreased average daytime bout number (p = 0.0038); (**G**) decreased average nighttime bout number (p = 0.0001); (**H**) increased average bout length (p=0.0002); (**I**) increased average daytime bout length (p = 0.0006); and (**J**) increased average nighttime bout length (p=0.0005). Each data point represents a single fly (n = number of flies); p-values were obtained by unpaired Student’s t-test with Welch’s correction (**D–G**) and Mann-Whitney U test (**H–J**). Averages are shown with error bars representing SEM. (See Table S1 for n and statistical analysis for each experiment; n.s.= p > 0.05, ** = p < 0.01, *** = p < 0.001, **** = p < 0.0001.)

The four biogenic amines modified by *ebony* (dopamine, serotonin, histamine, and octopamine) not only promote courtship and aggression, but also suppress sleep^40–45^. To test whether loss of *ebony* also influenced sleep, we used *Drosophila* Activity Monitors (DAMs) to measure and compare the sleep of *ebony* mutants and controls (Fig. 4A). Sleep duration was calculated by adding each subsequent minute after 5 min of inactivity, an analysis based on previous literature^46^, while a sleep bout is defined as a single, uninterrupted period of sleep (Fig. 4B). Sample activity and sleep plots are shown in Fig. 4C. We found that, while *ebony* mutants and controls exhibit similar average activity per hour (Fig. 4D), *ebony* mutants sleep more than controls (Fig. 4E). That is, *ebony* mutants exhibit increased activity during their wake time relative to controls (Fig. S4). To analyze sleep architecture, we quantified average sleep bout number and length during the day and night. *ebony* mutants exhibited fewer bouts of sleep during both daytime (Fig. 4F) and nighttime (Fig. 4G) and experienced an increased average sleep bout length (Fig. 4H) during both daytime (Fig. 4I) and nighttime (Fig. 4J). These results show that *ebony* mutants have fewer interrupted periods of sleep and more consolidated sleep architecture.

### Ebony in glia regulates aggression, courtship, and sleep

**Figure 5:**
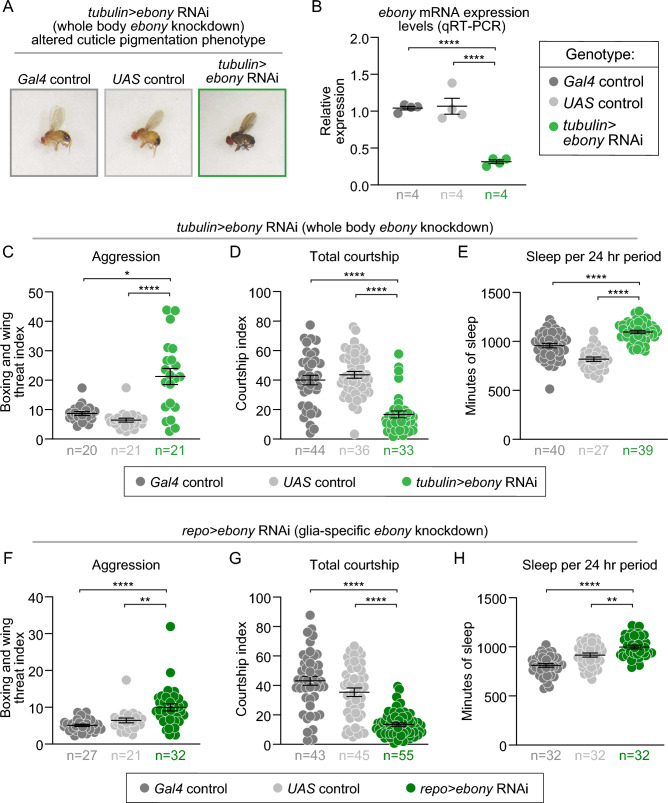
Whole body and glial-specific ebony RNAi knockdowns recapitulate ebony mutant behavioral phenotypes. (**A**) Images of *tubulin-Gal4* control (left), *UAS-ebony* RNAi control (center) and *tub-Gal4*>*UAS-ebony* RNAi flies (right) demonstrate the altered pigmentation phenotype resulting from ubiquitous ebony knockdown. (**B–H**) All *Gal4* controls are shown in dark gray, *UAS* controls in light gray, with *tubulin* > *ebony* RNAi in bright green (whole body), and *repo* > *ebony* RNAi in dark green (glia-specific). (**B**) *tubulin* > *ebony* RNAi flies exhibited less *ebony* mRNA expression than controls by qRT-PCR (p < 0.0001, n = number of biological replicates, 15 flies each). (**C–E**) Ubiquitous *ebony* RNAi knockdown led to: (**C**) increased boxing and wing threat aggressive behaviors, compared to *tubulin-Gal4* (p = 0.0225) and *UAS-ebony* RNAi (p < 0.0001) controls; (**D**) decreased total courtship, compared to *tubulin-Gal4* (p < 0.0001) and *UAS-ebony* RNAi (p < 0.0001) controls; and (**E**) increased sleep amount over a 24-hour period, compared to *tub-Gal4* (p < 0.0001) and *UAS-ebony* RNAi (p < 0.0001) controls (n = number of videos). (**F–H**) Glial-specific *ebony* RNAi knockdown led to: (**F**) increased boxing and wing threat aggressive behaviors, compared to *repo-Gal4* (p < 0.0001) and *UAS-ebony* RNAi (p = 0.0096) controls; (**G**) decreased total courtship, compared to *repo-Gal4* (p < 0.0001) and *UAS-ebony* RNAi (p < 0.0001) controls; and (**H**) increased sleep amount over a 24-hour period, compared to *repo-Gal4* (p < 0.0001) and *UAS-ebony* RNAi controls (p = 0.0099) (n = number of videos). p-values were obtained by ordinary one-way ANOVA (Dunnett’s multiple comparisons; B, E, H) and Kruskal-Wallis test with Dunn’s post hoc test (**C–D**, **F–G**). Averages are shown with error bars representing SEM. (See Table S1 for n and statistical analysis for each experiment; n.s.=p > 0.05, * = p < 0.05, ** = p < 0.01, **** = p < 0.0001.)

Because *ebony* is expressed in both glial cells and in the epidermal epithelium that produces the cuticle, we set out to test if all these observed behavioral changes in *ebony* mutants (increased aggression, decreased courtship, and increased sleep) are specifically due to Ebony’s function in glia. We first performed whole-body RNAi knock-down of *ebony* expression by driving *UAS-ebony* RNAi with a ubiquitous driver, *tubulin-Gal4*. Consistent with *ebony* mutants, whole-body RNAi-mediated knockdown of *ebony* caused the darkened cuticle characteristic of *ebony* mutants (Fig. 5A). To measure *ebony* expression in these flies, we performed qPCR analysis using whole flies and found significantly reduced mRNA levels in the whole-body *ebony* knockdown relative to both controls (Fig. 5B). Thus, *UAS-ebony* RNAi is an efficient tool to knock down *ebony* expression and test the role of *ebony* in glial cells to influence behavior.

To confirm that whole-body RNAi-mediated knockdown of *ebony* expression recapitulates the behavioral phenotypes of *ebony* mutants, we quantified aggression, courtship, and sleep in flies expressing *UAS-ebony* RNAi ubiquitously (using *tub-Gal4*)*.* Consistent with *ebony* mutants, whole-body RNAi knockdown of *ebony* (*tub-Gal4* > *UAS-ebony* RNAi) caused significant increases in aggression (as measured by boxing and wing threat) (Fig. 5C), decreases in courtship (Fig. 5D), and increases in sleep (Fig. 5E) relative to controls. These results confirmed that ubiquitous knockdown of *ebony* expression phenocopied *ebony* mutants.

To test if Ebony acts specifically in glial cells to affect behavior, we drove *UAS-ebony* RNAi with the well characterized, glia-specific *repo-Gal4* driver (*repo-Gal4* > *UAS-ebony* RNAi)^47^ and measured aggression, courtship, and sleep. Again, consistent with *ebony* mutants, glia-specific RNAi-mediated knockdown of *ebony* led to increased time spent exhibiting aggressive behaviors of boxing and wing threat (Fig. 5F), decreased time spent performing courtship behaviors (Fig. 5G), and increased sleep (Fig. 5H). Thus, taken together, our results show that Ebony protein in glia plays a major role in regulating these complex behaviors.

### *ebony* mutants have decreased neurotransmitter levels

**Figure 6:**
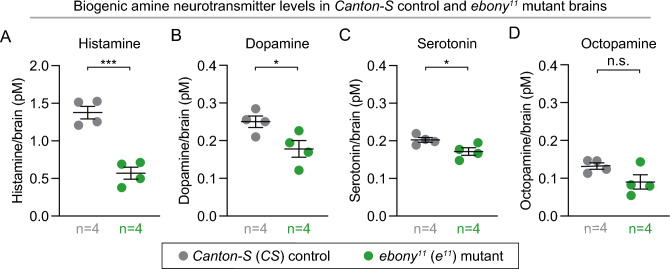
*ebony* mutant brains have decreased neurotransmitter levels relative to controls. (**A–D**) Each graph shows average neurotransmitter levels per brain of *ebony* mutants (green) and control flies (gray), measured by HPLC/MS (n = number of biological replicates, each consisting of 50 dissected brains with intact lamina). Relative to controls, *ebony* mutants had: (**A**) decreased histamine (p = 0.0004); (**B**) decreased dopamine (p=0.0410); (**C**) decreased serotonin (p = 0.0457); and (**D**) similar octopamine levels (p = 0.1084). p-values were obtained by unpaired Student’s t-test with Welch’s correction. Averages are shown with error bars representing SEM. (See Table S1 for n and statistical analysis for each experiment; n.s.= p > 0.05, * = p < 0.05, *** = p < 0.001.)

Loss of Ebony is predicted to increase levels of biogenic amine neurotransmitters, which would be consistent with the observed increase in hyper-aggressive behavior in *ebony* mutants, but not with decreased courtship behavior and increased sleep. To directly test how loss of Ebony affects biogenic amine transmitter levels, we used LC-MS/MS to measure levels of four unmodified (active, Fig. 1A) biogenic amine neurotransmitters (dopamine, histamine, serotonin, and octopamine) in dissected *ebony* and control brains with intact lamina. The *Drosophila* optic lobe contains a series of optic ganglia, the most peripheral of which is the lamina. We retained intact lamina because *ebony* is strongly expressed in glial cells of the lamina^11^. We found that *ebony* mutants exhibited decreased levels for three of the four biogenic amines assayed (Fig. 6A–D). Histamine, dopamine, and serotonin were reduced in *ebony* mutants compared to controls (Fig. 6A–C), with no significant difference in octopamine levels (Fig. 6D). Loss of histamine has been shown to lead to visual defects^48^. We assayed the specific aggressive behaviors of white-eyed *wCS* males and white-eyed *ebony* males (*w;;ebony*), known to have significant visual defects from the *white* mutation, to test if visual defects alone cause increased aggression (Fig. S5A–D). White-eyed *ebony* males exhibited significantly more boxing (Fig. S5A) and wing threat (Fig. S5B) behaviors, a similar level of lunging (Fig. S5C), and less chasing (Fig. S5D) compared to *wCS* controls. Comparing these aggressive behavioral indices to those of our red-eyed *Canton-S* control (Figs. 3; S3), *wCS* males exhibited similar or lower average aggressive behavior indices, particularly a lower average boxing index (Fig. S5A), while white-eyed *ebony* behavioral indices were comparable to those of red-eyed *ebony* males (Figs. 3; Fig. S3). Thus, impaired vision does not itself increase aggression. Overall, our finding of reduced biogenic amine levels in *ebony* mutants is consistent with the observed courtship and sleep phenotypes, but do not explain the elevated boxing and wing threat behaviors observed (Fig. 3B,C).

### The genotype of its opponent impacts *ebony*'s aggressive behavioral pattern

**Figure 7:**
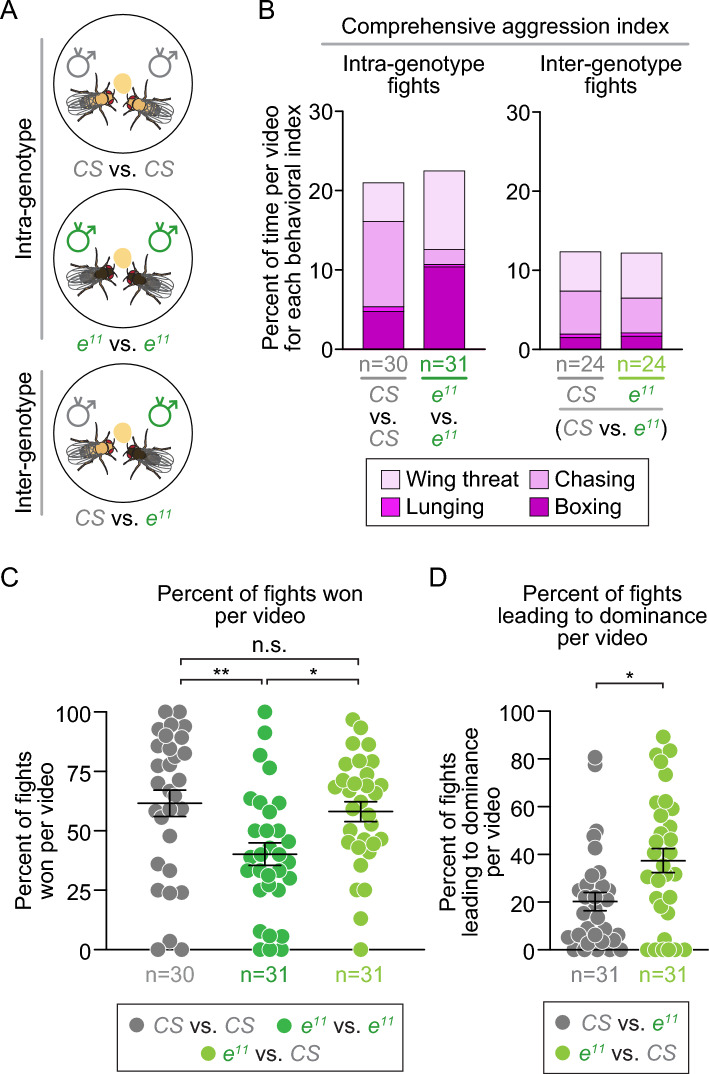
In inter-genotype fights, *ebony* do not display the aggression and dominance phenotypes seen in intra-genotype fights. (**A**) Schematic of intra-genotype fights (*CS* vs. *CS* or *ebony* vs. *ebony*) and inter-genotype fights (*CS* vs. *ebony*). (**B**) Stacked bars show the aggression index for specific aggressive behaviors in intra-genotype fights and inter-genotype fights (wing threat = light pink; chasing = pink; lunging = fuchsia; boxing = purple); note that the percentage of time per behavior per video is greater when quantifying two flies of the same genotype than each fly of different genotypes (n = number of videos). (**C**) While intra-genotype fights between *ebony* mutants (dark green) were less likely to result in a winner than intra-genotype fights between controls (gray) (p = 0.0057), inter-genotype fights between *ebony* mutants and controls (light green) were different from intra-genotype *ebony* fights (p = 0.0339) and as likely to result in a winner as fights between controls (p > 0.9999); n = number of videos. (**D**) In inter-genotype fights, *ebony* mutants won a greater number of fight bouts (p = 0.0175); n = number of videos. p-values were obtained by Kruskal-Wallis test with Dunn’s post hoc test (**C**) and Mann-Whitney U test (**D**). Averages are shown with error bars representing SEM. (See Table S1 for n and statistical analysis for each experiment; n.s.= p > 0.05, * = p < 0.05, ** = p < 0.01.)

To further explore *ebony*’s aggressive behaviors, we characterized *ebony*’s specific aggressive behavioral pattern and asked if their aggression patterns in intra-genotype fights (*CS* vs. *CS*, *ebony* vs. *ebony*) were similar for inter-genotype fights (*ebony* vs. *CS*) (Fig. 7A). Similar to the courtship index (CI) above, we determined a comprehensive aggression index (AI) as the percentage of frames per video that a male spent performing any of several aggressive behaviors (chasing, boxing, lunging, or wing threat) to examine overall patterns of behavior. When we viewed the profile of aggressive behavior in this way, we found that *ebony* mutants exhibited aggressive behaviors for the same amount of time as controls during both intra-genotype and inter-genotype fights, whether or not a hierarchy-based analysis was used (Figs. 7B; S6A; see Methods for description of hierarchy-based analysis). Though total aggression time was identical, *ebony* mutants had a distinct fighting profile in *ebony* vs. *ebony* fights, spending more time boxing and performing wing threat compared to controls (Fig. 7B, intra-genotype fights). This result was recapitulated for intra-genotype fights with RNAi-mediated knockdown of *ebony* in the whole body (*tubulin* > *ebony* RNAi) or glial cells (*repo* > *ebony* RNAi), which also exhibited elevated boxing and wing threat relative to respective controls (Fig. S6B). In contrast, in inter-genotype fights, we found a reduction in all four aggressive behaviors.

More strikingly, we observed that, in *ebony* vs. control fights, the fighting profile of *ebony* mutants became indistinguishable from controls (Fig. 7B, inter-genotype fights). When fighting controls, *ebony* mutants had similar total lunge count (Fig. S6C), lunge latency (Fig. S6D), and boxing, wing threat, lunging, and chasing indices (Fig. S6E–H) as controls. Taken together, these results suggest that *ebony* mutants exhibit a distinct pattern of "hyper-aggression", comprised of increased boxing and wing threat, mainly in intra-genotype fights with each other.

To further understand *ebony*’s hyper-aggression in *ebony* vs. *ebony* fights, we examined their dominance behavior, or their ability to “win” a fight bout. To score each video for a fly’s ability to establish dominance, we counted the percentage of fight bouts that occurred on food and led to a clear winner (i.e., a losing fly was chased/pushed off the food). A fight did not result in dominance if the flies ended a fight while both males remained on the food or left the food simultaneously. If a fight occurred off the food, then the fight was excluded from analysis. Because boxing is a behavior in which flies have reciprocally lunged at one another, essentially exhibiting equal aggression, we hypothesized that *ebony* mutants may have difficulty establishing a clear dominance relationship with each other in intra-genotype fights. Using this analysis, we found that 40% of *ebony* fights resulted in dominance compared to 62% of control fights (Fig. 7C). That is, *ebony* vs. *ebony* fights were less likely to have a clear winner than control fights, suggesting that *ebony* mutants have difficulty establishing dominance when fighting each other (Fig. 7C). Similarly, in the more ecologically relevant arena, *ebony* mutants in intra-genotype fights had reduced probability of establishing dominance compared to control intra-genotype fights; 42% of *ebony* videos exhibited dominance compared to 63% of control videos, though this was not significant (Fig. S6I).

In contrast, in *ebony* vs. *CS* control fights, we found that fights between *ebony* mutants and controls led to clear winners (58%), at a rate similar (62%) to control fights (Fig. 7C). In fights with *ebony* vs. controls, the winner was more likely to be *ebony* mutants, who won 38% of the time compared to 20% of the time for controls (Fig. 7D). Taken together, our results show that *ebony* mutants do not spend more time in total aggressive behavior than control males nor do they exhibit more aggressive behaviors like boxing when fighting with control males; nonetheless, they are more likely than controls to establish dominance in these intergenotype fights. We hypothesize that this preference for dominance leads to an escalation of aggressive behavior in *ebony* vs. *ebony* fights, in which flies had a reduced probability of establishing dominance and seemed to engage in reciprocal, prolonged aggression without retreating.

## Discussion

Here we set out to examine the behavior of *Drosophila* mutants lacking Ebony, a biogenic amine neurotransmitter recycling enzyme expressed in glia and epithelia. Previous literature from the 1960s and ′70s suggested that this mutant exhibited aberrant behavior but these changes in behavior had never been quantified systematically^3,27^. It was also unclear if *ebony* specifically in glia controls behavior as *ebony* is also expressed in other tissues, such as epithelial cells, which synthesize the cuticle, the site of cuticular hydrocarbons, which are important communication signals for *Drosophila* social behavior. We found, relative to controls, that *ebony* mutants exhibited increased hyper-aggressive behaviors, such as boxing, decreased courtship, and increased sleep; additionally, we determined that these changes in behavior are due to *ebony* expression specifically in glia. We quantified four common biogenic amines and substrates of Ebony in the brains of *ebony* mutants and controls. Contrary to expectation, we found that *ebony* mutants had lower levels of these neurotransmitters relative to controls. Finally, when we paired *ebony* and control males together for inter-genotype fights, we found that *ebony* mutants were no longer hyper-aggressive, though they were more likely to “win” a fight than controls. Thus, we hypothesize that *ebony* mutants paired together for intra-genotype fights use hyper-aggressive fighting tactics because they are less able to establish dominance.

Previous work characterizing *ebony* mutants’ behavior generated mixed results. The only published qualitative examination of aggression behavior for *ebony* mutants in two publications from Jacobs et al.^3,27^ observed that *ebony* males “fight more than do light males”^3^ and were more likely to be the territorial male when paired with control flies. Jacobs et al.^27^ also noted that *ebony* males emphasized tussling behavior. Here we show quantitative increases in specific types of aggressive behavior for *ebony* mutants relative to controls using automated analysis. Regarding *ebony* courtship, consistent with previous observations that *ebony* males have defective courtship behaviors^3,27,49,50^, we found that *e*^*11*^ mutants court significantly less than controls; one group reported increased wing extension duration in *ebony* mutants, which we did not observe^51^. Finally, another group previously found that *ebony*^*1*^ mutants exhibited decreased sleep^52^, in contrast to our finding of increased sleep in *ebony*^*11*^ mutants relative to controls (Fig. 2). We hypothesize that this and other possible differences in behavioral phenotype may be due to differences in *ebony* mutant alleles; as an example, one published study found that mutants containing different *ebony* alleles had different circadian locomotor phenotypes^53^. Because we could not validate two different stocks of the *e*^*1*^ mutants (BDSC#1658 and BDSC#8443) by PCR analysis and we were able to validate the *e*^*11*^ mutation both by PCR and western blot analysis, we used the *e*^*11*^ mutant or *ebony* RNAi for this work and caution others on using the *e*^*1*^ allele.

It should be noted that others have also previously measured dopamine and histamine levels in *ebony* mutants, with highly conflicting results. Different reports found higher, similar, or lower levels of different neurotransmitters; these contradictory results may be due to differences in *ebony* alleles, dissection technique, ages of flies, or quantification technique^54–58^. In some cases, *ebony* mutants also had extraneous and confounding mutations that affect neurotransmitter levels, such as *white* mutations^56,59^. To reduce these confounding variables, we used outcrossed (6 generations) and validated *e*^*11*^ mutants in an otherwise wild-type background, fully removed the cuticle from their brains, kept the lamina intact, age-matched to controls, used LC-MS/MS analysis (a highly sensitive and accurate technique), and measured all four major biogenic amines. Our definitive results settle this question and show that overall dopamine, histamine, and serotonin levels were significantly decreased in *ebony* mutants (Fig. 6A–C). Our results are further consistent with the prediction that loss of *ebony* should affect different biogenic amine neurotransmitters similarly.

Our finding of lower amine neurotransmitter levels in *ebony* mutants is consistent with two of the three behavioral phenotypes observed: decreased courtship and increased sleep. These two phenotypes are often associated with each other^8,9,31,40–45,54,60–63^ and also with lower amine neurotransmitter levels, especially dopamine^29,31,43–45,52,63^. These phenotypes are also consistent with low histamine levels, as histamine is required for proper vision in *Drosophila*^64,65^. *ebony* mutants, similar to white-eyed flies and other flies with visual defects^66^, exhibit less chasing and courtship behavior, likely because these require sighting of the target fly and recognition of other visual cues^50^.

The finding of decreased amine neurotransmitter levels in *ebony* mutants is more unexpected with regard to their aggressive behaviors; octopamine, dopamine, and serotonin all positively regulate *Drosophila* aggression^4,31–34,67^. It should be noted that our analysis quantified total neurotransmitter levels, not extracellular neurotransmitter levels. The specific molecular mechanism by which decreased neurotransmitter levels leads to increased aggression remains unclear. One possibility is an effect on pheromone secretion or reception. *ebony* mutants have been shown to exhibit differences in cuticular hydrocarbons^68^, pheromones that modulate fly interactions. Cuticular hydrocarbons are made in large secretory cells called oenocytes that are known to express *ebony*^69^. Because we showed here that changes in sleep, courtship, and aggression in *ebony* mutants are due to loss of *ebony* specifically in glia, our work raises the exciting possibility that *ebony* mutants may be the first known example of altered glia to oenocyte signaling and regulation. Determining whether and how loss of *ebony* in glia alters cuticular hydrocarbons to increase aggression will be an interesting question for future investigation.

One major finding from this work came from our analysis of *ebony* vs. control fights: we found that *ebony* males are more likely to establish dominance and win fights against control males, suggesting a basis for their hyper-aggressive behavior in intra-genotype fights. In *Drosophila* aggression, dominance can be observed in the fly that maintains its position on the food, or the “territory,” over which the males are fighting^70^. Previous work had suggested that *ebony* mutants are highly territorial, tending to defend and stay close to their territory (food) more than controls^27^. Our dominance analysis is consistent with this conclusion and further supports the hypothesis that *ebony* mutants tend toward dominance and engage in hyper-aggressive behavior when they are also unable to establish dominance. That is, *ebony* vs. *ebony* fights tend to escalate as neither fly is willing to concede.

It is worth noting that glial Ebony may play a developmental role in neuronal circuit formation or in building brain structures^71^ required for normal adult behavior. That is, because *ebony* mutants lack Ebony for their entire lifespan, including development, loss of Ebony in glia during development, as opposed to or in addition to loss of Ebony during adulthood, could alter neuronal structure, circuit formation, or neuronal activity/function and thereby drive adult behavioral phenotypes. Conditional knockdown of glial *ebony* only during development or only during adulthood and assaying for subsequent behaviors would clarify the time window during which loss of *ebony* has greatest impact on adult behavior. Consistent with our work, mouse mutants genetically lacking MAO have also been shown to exhibit increased aggressive behavior^72–76^. Moreover, specific polymorphisms in both MAO and COMT are associated with aggressive behavior^77–81^. Thus, our results are consistent with work in mammals and suggest that chronic inhibition of an inactivator of neurotransmitters may lead to decreased neurotransmitter levels and unanticipated effects in genetically predisposed individuals, such as increased aggression in the context of a fight between two dominance-driven opponents. Given the widespread use of pharmacological inhibitors of MAO^22^ and COMT^82^, understanding the long-term effects of inhibiting these enzymes with chronic treatment or during development (in utero, childhood, or adolescence) may have a broad and significant impact.

## Materials and methods

### Fly strains

**Table:** 

Genotype	Source	Identifiers
*CS;;ebony* (*ebony* mutant)	Bloomington *Drosophila* Stock Center, outcrossed by Shirasu-Hiza Lab	BDSC #497
*CS;CS;CS* (background of *wCS* strain)	Shirasu-Hiza Lab	
*UAS-ebony* RNAi	Bloomington *Drosophila* Stock Center	BDSC #28,612
Control for *UAS-ebony* RNAi	Bloomington *Drosophila* Stock Center	BDSC #36,303
*wCS;;tubulin-Gal4*	Shirasu-Hiza Lab	
*wCS;;repo-Gal4*	Bloomington *Drosophila* Stock Center, outcrossed by Shirasu-Hiza Lab	BDSC #7415

### ebony Mutant selection

While the *e*^*1*^ mutant is more commonly used in *ebony* research, we chose to work with *e*^*11*^ because we could not molecularly validate two independent *e*^*1*^ stocks obtained from the Bloomington *Drosophila* Stock Center (BDSC #1658 and BDSC #8443). According to Miller et al.^83^, *e*^*1*^ mutants are expected to have a transposable element inserted in exon 2 (FlyBase)^84^, but amplifying the region around the expected insertion site produced a PCR band that was similar in size to that of the wild-type fly. The *e*^*11*^ mutant, on the other hand, produced the expected PCR band containing a ~ 500 bp deletion. Our results are consistent with a previously published report^28^, which found the expected deletion in the *e*^*11*^ mutant through PCR and DNA sequencing and a wildtype-sized exon 2 region in the *e*^*1*^ mutant. Those authors, like us, also could not sequence that portion of the *e*^*1*^ allele and suggested that an unknown inversion or insertion may have occurred in exon 3.

### Sleep and locomotor activity

6 to 8-day-old male flies entrained on a 12:12 Light–Dark cycle were placed in individual 5 mm plastic tubes containing food. Tubes were placed in TriKinetics *Drosophila* Activity Monitors (DAMs)^85^ to record their locomotor activity for 5 days in 12:12 Light:Dark (LD). Beam break data was grouped into 1-min bins using DAM File Scan, and pySolo^86^ (Python-based software) was used to analyze sleep architecture and waking activity. Sleep was defined as a period of at least 5 min of inactivity^46^.

### Courtship assay

Virgin males were collected from crosses within 6 h of eclosion, stored individually in vials for 6–8 days, and entrained on a 12:12 LD cycle. We used 16 mm diameter testing chambers (Greiner Bio-One 24-well no bottom plates) coated with Insect-a-Slip (BioQuip) (also referred to as Fluon) to prevent wall climbing. Two plates were stacked on top of each other with a plastic divider separating them, and the bottom plate was taped underneath with a plastic divider to prevent flies from escaping. Sexually unreceptive *wCS* females (mated within the last 24 h) were aspirated without anesthesia into the central eight wells of the bottom plate and males were aspirated without anesthesia into the corresponding chambers of the top plate. The plastic divider was removed and the male was dropped into the chamber with the female and videotaped for 10 min using Supereyes B003 + cameras and iSpy software to record courtship behavior towards the female at a frame rate consistent between control and experimental flies within each experiment, of either 10 or 20 frames per second (depending on the computer used for acquisition). Because courtship index represents frames of activity divided by total number of frames, differences in frame rate are normalized. We excluded videos with copulation from our analysis unless otherwise specified (i.e., when examining rates of copulation).

### Aggression assay

The same virgin collection conditions and testing chambers were used as for the courtship assay (see above). Using a 1 mL syringe 2–3 h before testing, a single drop of food (Glucose – Archon Scientific) was placed in the center of each well in the bottom plate. One male from each pair was aspirated without anesthesia into one of the central eight wells of the bottom plate. The remaining male from each pair was aspirated without anesthesia into the corresponding well of the top plate. The plastic divider was removed so that the male from the top plate dropped into the bottom well. The males were videotaped for 10 min using Supereyes B003 + cameras and iSpy software to record aggressive behavior at a frame rate consistent between control and experimental flies within each experiment, of either 10 or 20 frames per second (depending on the computer used for acquisition). Because aggression index represents frames of activity divided by total number of frames, differences in frame rate are normalized. Aggression assays performed with the more ecologically relevant setup were done as described in Fernandez et al.^39^.

### Quantification of courtship and aggression behaviors

The CalTech FlyTracker and JAABA softwares were used to automatically quantify the courtship index (CI)/aggression index (AI), or percent of frames spent courting/fighting in a 10-min period, for each male. The Caltech FlyTracker automatically generates data for each video frame such as the location of the male and female, the angle of the wings, the velocity of the flies, etc. We visually inspected each video to ensure tracking accuracy; if a video had tracking errors > 5% of frames we discarded the video. We trained the machine learning algorithm (JAABA) to quantify wing extension, chasing, and attempted mounting behaviors for courtship frame by frame based on the data from the Caltech FlyTracker. We trained the classifiers to detect boxing, wing threat, lunging, and chasing behaviors for aggression in the same way. Because two or more behaviors can co-occur simultaneously (such as wing extension and chasing in courtship, and boxing and wing threat in aggression), it is typical in the field to use a hierarchical code that would not double count frames as “courtship” or “aggression” when computing total courtship and aggression levels (i.e., Fig. 3B and S5A). Our courtship code gives greatest priority to wing extension, followed by chasing and attempted mounting; that is, if wing extension and chasing co-occur in a video frame, JAABA annotates the frame for wing extension only. Our aggression hierarchy gives precedence to boxing, followed by wing threat, chasing, and lunging. For non-hierarchical analysis of total time spent exhibiting aggressive behaviors, which we quantified in Figs. 7B and S5B, we computed the behavioral index for each behavior separately in JAABA and summed.

For aggression videos involving intra-genotype pairing, the aggression index was analyzed for the dominant fly of each fight, determined by which fly exhibited more offensive behavior. In videos with inter-genotype pairing, we manually checked each annotated videos to ensure Caltech FlyTracker accurately tracks the *ebony* mutant and control fly, which have distinct cuticle colors that can be discerned in our testing chambers.

To assess dominance, we counted the number of fight bouts occurring on food, which can involve a broad range of aggressive behaviors described by Chen et al.^37^, that led to a clear winner. The fly that successfully chases or nudges the opponent fly off the food is considered the winner of that fight encounter. A fight did not result in dominance if the flies ended a fight and both males remained on the food or left the food simultaneously. We excluded from this analysis any fights that did not occur on the food. Dominance in the more ecologically relevant arena was scored as described in Penn et al.^87^.

### Statistical analysis

We assessed the normality of our data using the D’Agostino-Pearson normality test and the Shapiro–Wilk normality test, which have great power properties over a variety of different statistical distributions^88^. For data sets that passed both normality tests, we used the unpaired Student’s t-test with Welch’s correction when comparing two groups and the one-way ANOVA (Dunnett’s multiple comparisons) when comparing three or more groups. For data sets that failed either one of the normality tests, we used the Mann–Whitney U test when comparing two groups and the Kruskal–Wallis test with Dunn’s post hoc test when comparing three or more groups. For binary comparisons, we used Fisher’s exact test. See Table S1 for the n, statistical analyses, and justification for statistics for all experiments. All plotted values represent means, with error bars representing SEM.

### qPCR

7-day-old male flies previously entrained to 12:12 LD were snap-frozen in liquid nitrogen and stored at −80 °C. RNA was extracted from 15 whole flies for each of 4 biological replicates per genotype with TRIzol (Invitrogen) following the manufacturer’s protocol. Samples were treated with DNaseI (Invitrogen), then heat inactivated. cDNA was synthesized by Revertaid First Strand cDNA Synthesis Kit (Thermo Scientific). PowerUp SYBR Mastermix (Applied Biosystems) was used to perform qRT-PCR using a CFXConnect thermal cycler (BioRad). Primer efficiency and relative quantification of transcripts were determined using a standard curve of serial diluted cDNA. Transcripts were normalized using *Actin5C* as a reference gene.

Primer Sequences:

*ebony*-fwd-GTCCGAAGTGGAGAAGAACG

*ebony*-rev-TCTGTGCTACCATGCTGGTC

*Actin5C*-fwd-TTGTCTGGGCAAGAGGATCAG.

*Actin5C*-rev-ACCACTCGCACTTGCACTTTC.

### Western blot

Whole-body lysates of 10-day-old male flies (15 flies/sample) were separated by SDS-PAGE on a Tris–Acetate 3–8% gel (NuPAGE, Thermo Scientific), and transferred to a PVDF membrane (Immobilon, Thermo Scientific). The membrane was probed with rabbit polyclonal antibody against Ebony at 1:1000 in 3% BSA (kind gift from Dr. Wittkopp)^89^. HRP-conjugated anti-rabbit IgG antibody at 1:2000 in 3% BSA was used for signal detection (Cell Signaling, 7074). ECL chemiluminescence reagent (Pierce) was used to visualize horseradish peroxidase activity and detected by CCD camera using a BioRAD Image Station.

### Quantification of neurotransmitters

*ebony* and *CS* brains, with laminas intact, were dissected in PBS on ice. 50 brains were dissected per sample, with four samples collected for each genotype (200 brains total/genotype). Before freezing, samples were gently spun down and excess PBS was pipetted off. Samples were stored into a −80 °C freezer, then shipped on dry ice to Children’s Hospital of Philadelphia Metabolomic Core for analysis.

Dopamine (DOP), serotonin (SE), octopamine (OCTOP), and histamine (HA) levels were determined using LC–MS/MS the Agilent 1260 Infinity Triple Quad 6410B mass spectrometry (MS) coupled with liquid chromatography (LC) system^90,91^. Both isotope dilution methodology and standard curve were used to determine neurotransmitter levels. To each sample of brain tissue extract, 2 pM of D4 labeled HA, SR, OCTOP and D3-DOP were added. Then, 160 µL of ethanol and 40 µL of pyridine were added. Derivatization was performed by adding 50 µL of ethyl chloroformate and shaking gently the vial until bubbles were gone. Derivatized neurotransmitters were extracted with ethyl acetate, and the organic phase was dried down and reconstituted in 100 µL of 0.1% of formic acid, spun at 14,000 rpm and transferred into injection vials. 25 µL of the derivatized sample were injected into the LC-MS system. For chromatographic separation, we used Agilent Poroshell 120 column (EC-C18), 3 × 100 µM. Solution A was 0.1% of formic acid and Solution B acetonitrile with 0.1% of formate and 0.005% of tri-fluoro-acetate (TFA). Flow gradient started with 50% of solution B, and then 60% after 3 min, 70%, after 5 min and 100% at 6–15 min. Precursor-product used for each compound was as follows: HA 256-138 and D4-HA 260-142 MRM; OCTOP 280-91 and D4 OCTOP 284-95; SE, 321-203 and D4-SE 325-207 MRM; DOP 370-252 and D3-DOP 373-255 MRM. Concentration of each compound in the sample was calculated based on isotopic enrichment as described^92^ and normalized to the amount of cellular protein/sample. Simultaneously, the concentration of each compound was verified by using standard curve prepared with a known concentration of each neurotransmitter ranging from 2 to 10 pM/mL and determination of the area under the peak of each compound.

### Supplementary Information

Supplementary Legends.

Supplementary Figure S1.

Supplementary Figure S2.

Supplementary Figure S3.

Supplementary Figure S4.

Supplementary Figure S5.

Supplementary Figure S6.

Supplementary Table S1.

## Data Availability

The authors declare that all data supporting the findings of this study are available, including replicate experiments, and will be made available upon reasonable request to the corresponding author, Dr. Mimi Shirasu-Hiza.

## References

[1] Perez MM, Schachter J, Berni J, Quesada-Allue LA (2010). The enzyme NBAD-synthase plays diverse roles during the life cycle of *Drosophila melanogaster*. J. Insect Physiol..

[2] Richardt A (2003). Ebony, a novel nonribosomal peptide synthetase for beta-alanine conjugation with biogenic amines in *Drosophila*. J. Biol. Chem..

[3] Jacobs ME (1960). Influence of light on mating of *Drosophila melanogaster*. Ecology.

[4] Kravitz EA, Fernandez MP (2015). Aggression in *Drosophila*. Behav. Neurosci..

[5] Zhang W, Guo C, Chen D, Peng Q, Pan Y (2018). Hierarchical control of *Drosophila* sleep, courtship, and feeding behaviors by male-specific P1 neurons. Neurosci. Bull..

[6] Koganezawa M, Kimura K, Yamamoto D (2016). The neural circuitry that functions as a switch for courtship versus aggression in *Drosophila* males. Curr. Biol..

[7] Zwarts L, Versteven M, Callaerts P (2012). Genetics and neurobiology of aggression in *Drosophila*. Fly.

[8] Zhou C (2012). Molecular genetic analysis of sexual rejection: Roles of octopamine and its receptor OAMB in *Drosophila* courtship conditioning. J. Neurosci..

[9] Zhang SX, Rogulja D, Crickmore MA (2016). Dopaminergic circuitry underlying mating drive. Neuron.

[10] Suh J, Jackson FR (2007). *Drosophila* ebony activity is required in glia for the circadian regulation of locomotor activity. Neuron.

[11] Richardt A, Rybak J, Stortkuhl KF, Meinertzhagen IA, Hovemann BT (2002). Ebony protein in the *Drosophila* nervous system: optic neuropile expression in glial cells. J. Comp. Neurol..

[12] Hovemann BT (1998). The *Drosophila* ebony gene is closely related to microbial peptide synthetases and shows specific cuticle and nervous system expression. Gene.

[13] Ng FS, Tangredi MM, Jackson FR (2011). Glial cells physiologically modulate clock neurons and circadian behavior in a calcium-dependent manner. Curr. Biol..

[14] Freeman MR, Doherty J (2006). Glial cell biology in *Drosophila* and vertebrates. Trends Neurosci..

[15] Volkenhoff A (2015). Glial glycolysis is essential for neuronal survival in *Drosophila*. Cell Metab..

[16] Logan MA (2017). Glial contributions to neuronal health and disease: new insights from *Drosophila*. Curr. Opin. Neurobiol..

[17] Semenoff D, Kimelberg HK (1985). Autoradiography of high affinity uptake of catecholamines by primary astrocyte cultures. Brain Res..

[18] Grosjean Y, Grillet M, Augustin H, Ferveur JF, Featherstone DE (2008). A glial amino-acid transporter controls synapse strength and courtship in *Drosophila*. Nat. Neurosci..

[19] Rival T (2006). Physiological requirement for the glutamate transporter dEAAT1 at the adult *Drosophila* neuromuscular junction. J. Neurobiol..

[20] Jackson FR, Haydon PG (2008). Glial cell regulation of neurotransmission and behavior in *Drosophila*. Neuron Glia Biol..

[21] Bilder RM, Volavka J, Lachman HM, Grace AA (2004). The catechol-O-methyltransferase polymorphism: Relations to the tonic-phasic dopamine hypothesis and neuropsychiatric phenotypes. Neuropsychopharmacology.

[22] Yeung AWK, Georgieva MG, Atanasov AG, Tzvetkov NT (2019). Monoamine oxidases (MAOs) as privileged molecular targets in neuroscience: Research literature analysis. Front. Mol. Neurosci..

[23] Hull EM, Muschamp JW, Sato S (2004). Dopamine and serotonin: Influences on male sexual behavior. Physiol. Behav..

[24] Connor DF, Harrison RJ, Melloni RH (1998). Biogenic amines and the psychopharmacology of aggression. Expert Opin. Ther. Pat..

[25] Eyjolfsdottir, E. *et al.* 772–787 (Springer International Publishing).

[26] Kabra M, Robie AA, Rivera-Alba M, Branson S, Branson K (2013). JAABA: interactive machine learning for automatic annotation of animal behavior. Nat. Methods.

[27] Jacobs ME (1978). Influence of beta-alanine on mating and territorialism in *Drosophila melanogaster*. Behav. Genet..

[28] Pérez, M. M., Rossi, F., Quesada-Allué, L. A., Comparison of ebony gene from three ebony mutants. *Drosophila Inf. Serv.* **97**, 30–32 (2014).

[29] Pooryasin A, Fiala A (2015). Identified serotonin-releasing neurons induce behavioral quiescence and suppress mating in *Drosophila*. J. Neurosci..

[30] Liu T (2008). Increased dopamine level enhances male-male courtship in *Drosophila*. J. Neurosci..

[31] Andrews JC (2014). Octopamine neuromodulation regulates Gr32a-linked aggression and courtship pathways in *Drosophila* males. PLoS Genet..

[32] Alekseyenko OV, Chan YB, Li R, Kravitz EA (2013). Single dopaminergic neurons that modulate aggression in *Drosophila*. Proc. Natl. Acad. Sci. U S A.

[33] Dierick HA, Greenspan RJ (2007). Serotonin and neuropeptide F have opposite modulatory effects on fly aggression. Nat Genet.

[34] Hoyer SC (2008). Octopamine in male aggression of *Drosophila*. Curr Biol.

[35] Everaerts C, Farine JP, Cobb M, Ferveur JF (2010). Drosophila cuticular hydrocarbons revisited: mating status alters cuticular profiles. PLoS One.

[36] Ejima A (2007). Generalization of courtship learning in *Drosophila* is mediated by cis-vaccenyl acetate. Curr. Biol..

[37] Chen S, Lee AY, Bowens NM, Huber R, Kravitz EA (2002). Fighting fruit flies: a model system for the study of aggression. Proc. Natl. Acad. Sci. U S A.

[38] Chowdhury B, Wang M, Gnerer JP, Dierick HA (2021). The Divider Assay is a high-throughput pipeline for aggression analysis in *Drosophila*. Commun. Biol..

[39] Fernandez MP (2010). Pheromonal and behavioral cues trigger male-to-female aggression in *Drosophila*. PLoS Biol..

[40] Ly S, Pack AI, Naidoo N (2018). The neurobiological basis of sleep: Insights from *Drosophila*. Neurosci. Biobehav. Rev..

[41] Oh Y, Jang D, Sonn JY, Choe J (2013). Histamine-HisCl1 receptor axis regulates wake-promoting signals in *Drosophila melanogaster*. PLoS One.

[42] Crocker A, Shahidullah M, Levitan IB, Sehgal A (2010). Identification of a neural circuit that underlies the effects of octopamine on sleep:wake behavior. Neuron.

[43] Yuan Q, Joiner WJ, Sehgal A (2006). A sleep-promoting role for the *Drosophila* serotonin receptor 1A. Curr. Biol..

[44] Crocker A, Sehgal A (2008). Octopamine regulates sleep in *Drosophila* through protein kinase A-dependent mechanisms. J. Neurosci..

[45] Kume K, Kume S, Park SK, Hirsh J, Jackson FR (2005). Dopamine is a regulator of arousal in the fruit fly. J. Neurosci..

[46] Huber R (2004). Sleep homeostasis in *Drosophila melanogaster*. Sleep.

[47] Awasaki T, Ito K (2004). Engulfing action of glial cells is required for programmed axon pruning during *Drosophila* metamorphosis. Curr. Biol..

[48] Borycz J, Borycz JA, Edwards TN, Boulianne GL, Meinertzhagen IA (2012). The metabolism of histamine in the *Drosophila* optic lobe involves an ommatidial pathway: Beta-alanine recycles through the retina. J. Exp. Biol..

[49] Rendel JM (1951). Mating of ebony vestigial and wild type *Drosophila-melanogaster* in light and dark. Evolution.

[50] Crossley S, Zuill E (1970). Courtship behaviour of some *Drosophila-melanogaster* mutants. Nature.

[51] Kyriacou CP, Burnet B, Connolly K (1978). The behavioural basis of overdominance in competitive mating success at the ebony locus of *Drosophila melanogaster*. Anim. Behav..

[52] Rossi FA, Bochicchio PA, Quesada-Allué LA, Pérez MM (2015). N-β-alanyldopamine metabolism, locomotor activity and sleep in *Drosophila melanogaster* ebony and tan mutants. Physiol. Entomol..

[53] Newby LM, Jackson FR (1991). Drosophila-ebony mutants have altered circadian activity rhythms but normal eclosion rhythms. J. Neurogenet..

[54] Ramadan H, Alawi AA, Alawi MA (1993). Catecholamines in *Drosophila melanogaster* (wild type and ebony mutant) decuticalarized retinas and brains. Cell Biol. Int..

[55] Gruntenko N (2004). The effect of mutations altering biogenic amine metabolism in *Drosophila* on viability and the response to environmental stresses. Arch Insect. Biochem. Physiol..

[56] Hodgetts RB, Konopka RJ (1973). Tyrosine and catecholamine metabolism in wild-type *Drosophila melanogaster* and a mutant, ebony. J. Insect Physiol..

[57] Borycz J, Borycz JA, Loubani M, Meinertzhagen IA (2002). tan and ebony genes regulate a novel pathway for transmitter metabolism at fly photoreceptor terminals. J. Neurosci..

[58] Denno ME, Privman E, Borman RP, Wolin DC, Venton BJ (2016). Quantification of histamine and carcinine in *Drosophila melanogaster* tissues. ACS Chem. Neurosci..

[59] Borycz J, Borycz JA, Kubow A, Lloyd V, Meinertzhagen IA (2008). *Drosophila* ABC transporter mutants white, brown and scarlet have altered contents and distribution of biogenic amines in the brain. J. Exp. Biol..

[60] Sitaraman D, Aso Y, Rubin GM, Nitabach MN (2015). Control of sleep by dopaminergic inputs to the *Drosophila* mushroom body. Front. Neural Circuits.

[61] Ueno T (2012). Identification of a dopamine pathway that regulates sleep and arousal in *Drosophila*. Nat. Neurosci..

[62] Lim J (2018). The mushroom body D1 dopamine receptor controls innate courtship drive. Genes Brain Behav..

[63] Erion R, DiAngelo JR, Crocker A, Sehgal A (2012). Interaction between sleep and metabolism in Drosophila with altered octopamine signaling. J. Biol. Chem..

[64] Hardie RC (1987). Is histamine a neurotransmitter in insect photoreceptors?. J. Comp. Physiol. A.

[65] Sarthy PV (1991). Histamine: A neurotransmitter candidate for *Drosophila* photoreceptors. J. Neurochem..

[66] Xiao C, Qiu S, Robertson RM (2017). The white gene controls copulation success in *Drosophila* melanogaster. Sci. Rep..

[67] Asahina K (2017). Neuromodulation and strategic action choice in *Drosophila* aggression. Annu. Rev. Neurosci..

[68] Massey JH (2019). Pleiotropic effects of ebony and tan on pigmentation and cuticular hydrocarbon composition in *Drosophila melanogaster*. Front. Physiol..

[69] Huang K, Liu Y, Perrimon N (2022). Roles of insect oenocytes in physiology and their relevance to human metabolic diseases. Front. Insect Sci..

[70] Kaufmann JH (1983). On the definitions and functions of dominance and territoriality. Biol. Rev..

[71] Ullian EM, Christopherson KS, Barres BA (2004). Role for glia in synaptogenesis. Glia.

[72] Mejia JM, Ervin FR, Baker GB, Palmour RM (2002). Monoamine oxidase inhibition during brain development induces pathological aggressive behavior in mice. Biol. Psychiatry.

[73] Frau R, Pardu A, Godar S, Bini V, Bortolato M (2022). Combined antagonism of 5-HT(2) and NMDA receptors reduces the aggression of monoamine oxidase a knockout mice. Pharmaceuticals.

[74] Magwai T, Xulu KR (2022). Physiological genomics plays a crucial role in response to stressful life events, the development of aggressive behaviours, and post-traumatic stress disorder (PTSD). Genes.

[75] Scott AL, Bortolato M, Chen K, Shih JC (2008). Novel monoamine oxidase A knock out mice with human-like spontaneous mutation. NeuroReport.

[76] Shih JC (1999). Ketanserin and tetrabenazine abolish aggression in mice lacking monoamine oxidase A. Brain Res..

[77] Qayyum A (2015). The role of the catechol-o-methyltransferase (COMT) GeneVal158Met in aggressive behavior, a review of genetic studies. Curr. Neuropharmacol..

[78] Rujescu D, Giegling I, Gietl A, Hartmann AM, Moller HJ (2003). A functional single nucleotide polymorphism (V158M) in the COMT gene is associated with aggressive personality traits. Biol. Psychiat..

[79] Volavka J, Bilder R, Nolan K (2004). Catecholamines and aggression—The role of COMT and MAO polymorphisms. Youth Violence: Sci. Approaches Prev..

[80] Tosato S (2011). Effect of COMT genotype on aggressive behaviour in a community cohort of schizophrenic patients. Neurosci. Lett..

[81] Alia-Klein N (2008). Brain monoamine oxidase a activity predicts trait aggression. J. Neurosci..

[82] Fabbri M, Ferreira JJ, Rascol O (2022). COMT inhibitors in the management of parkinson's disease. CNS Drugs.

[83] Miller DE, Cook KR, Arvanitakis AV, Hawley RS (2016). Third chromosome balancer inversions disrupt protein-coding genes and influence distal recombination events in *Drosophila melanogaster*. G3-Genes Genomes Genetics.

[84] Larkin A (2021). FlyBase: Updates to the *Drosophila melanogaster* knowledge base. Nucleic Acids Res.

[85] Rosato E, Kyriacou CP (2006). Analysis of locomotor activity rhythms in *Drosophila*. Nat. Protoc..

[86] Gilestro GF, Cirelli C (2009). pySolo: A complete suite for sleep analysis in *Drosophila*. Bioinformatics.

[87] Penn JK, Zito MF, Kravitz EA (2010). A single social defeat reduces aggression in a highly aggressive strain of *Drosophila*. Proc. Natl. Acad. Sci. U S A.

[88] Yap BW, Sim CH (2011). Comparisons of various types of normality tests. J. Stat. Comput. Simul..

[89] Wittkopp PJ, True JR, Carroll SB (2002). Reciprocal functions of the *Drosophila* yellow and ebony proteins in the development and evolution of pigment patterns. Development.

[90] Castillero, E. *et al.* Decreased serotonin transporter activity in the mitral valve contributes to progression of degenerative mitral regurgitation. *Sci. Transl. Med.* (in press). 10.1126/scitranslmed.adc9606 PMC9896655 36599005

[91] van de Merbel NC (2011). Quantitative determination of free and total dopamine in human plasma by LC-MS/MS: The importance of sample preparation. Bioanalysis.

[92] Weinberg JM, Venkatachalam MA, Roeser NF, Nissim I (2000). Mitochondrial dysfunction during hypoxia/reoxygenation and its correction by anaerobic metabolism of citric acid cycle intermediates. Proc. Natl. Acad. Sci. U S A.

