# Supplementary material for: Drosophila mutants lacking the glial neurotransmitter-modifying enzyme Ebony exhibit low neurotransmitter levels and altered behavior
Source: Sci Rep. 2023 Jun 27;13:10411. doi: 10.1038/s41598-023-36558-7 (PMC10300103; doi:10.1038/s41598-023-36558-7)
Supplement: Supplementary file 1 — Supplementary Legends. [file 41598_2023_36558_MOESM1_ESM.docx]

**Figure S1. Further validation of *ebony^11^* and automated behavior ground-truthing analysis.** (A) Images of control (left) and *ebony^11^* (right) flies demonstrate the altered pigmentation phenotype in *ebony^11^* mutants. (B) Relative *ebony* mRNA levels measured by qRT-PCR. *ebony^11^* mutants (green) exhibited more *ebony* expression than controls (gray, p=0.0001) (n=number of biological repeats, each containing 15 flies). (C) Whole western blot showing that *ebony^11^* mutants lack Ebony protein; red dashed line indicates section shown in Fig. 1C. (D) Table of ground truthing results used to validate the accuracy of JAABA classifiers: courtship (wingsong, chasing, and attempted mounting, in blue) and aggression (boxing, wing threat, chasing, and lunging, in pink). p-values were obtained by an unpaired Student’s t-test with Welch’s correction. Averages are shown with error bars representing SEM. (See Table S1 for n and statistical analysis for each experiment; ***=p<0.001.)

**Figure S2. *ebony* mutants court virgin females less than controls. (**A) Schematic of courtship assay using *wCS* virgin females. (B-C) When courting virgin females, *ebony* mutants (green) exhibited, relative to controls (gray): (B) decreased total courtship (p<0.0001); and (C) increased latency to first courting event (p<0.0001). (D) When courting *wCS* virgin females, *ebony* mutants (green) copulated less frequently (p<0.0001) than controls (gray). n represents the number of videos, each containing one male; p-values were obtained by Mann-Whitney U test (B-C) and Fisher’s exact test (D). Averages are shown with error bars representing SEM. (See Table S1 for n and statistical analysis for each experiment; ****=p<0.0001.)

**Figure S3. *ebony* mutants box more and chase less, but have the same latency to lunge as controls in a more ecologically relevant arena.** (A-B) In intra-genotype fights, *ebony* mutants (green), compared to controls (gray), displayed: (A) a greater incidence of boxing behavior (p=0.0159); and (B) less chasing behavior (p<0.0001); each data point represents a video with two males. (C) In a more ecologically relevant arena, *ebony* mutants (green) showed a latency to lunge similar to controls (gray, p=0.1962). n=number of videos; p-values were obtained by Fisher’s exact test (A), Mann-Whitney U test (B), and unpaired Student’s t-test with Welch’s correction (C). Averages are shown with error bars representing SEM. (See Table S1 for n and statistical analysis for each experiment; n.s.=p>0.05, ****=p<0.0001.)

**Figure S4. *ebony* mutants are more active when awake than controls.** (A) *ebony* mutants (green) exhibited increased activity per waking minute (p<0.0001) compared to controls (gray). Each data point represents a single fly (n=number of flies); p-values were obtained by unpaired Student’s t-test with Welch’s correction. Averages are shown with error bars representing SEM. (See Table S1 for n and statistical analysis for each experiment; ****=p<0.0001.)

**Figure S5. Aggressive behavior of visually-impaired white-eyed *ebony* males are similar to red-eyed *ebony*.** Behavioral indices were quantified for *wCS* and *w;;ebony* male intra-genotype fights for (A) boxing, (B) wing threat, (C) lunging, and (D) chasing behaviors. *w;;ebony* mutants exhibited: (A) increased boxing behavior (p<0.0001); (B) increased wing threat (p<0.0001); (C) no difference in lunging behavior; (D) and decreased chasing (p<0.0001); each data point represents a video of two flies (n=number of videos). Note that mean indices for *wCS* males (boxing 2.29, wing threat 3.2, lunging 0.09, chasing 3.53) were lower than those observed for red-eyed control males in Fig. 3, S3 (boxing 4.27, wing threat 4.92, lunging 0.26, chasing 9.93), and mean indices of white-eyed *ebony* males (boxing 8.47, wing threat 9.06, lunging 0.13, chasing 1.23) were similar to those observed for red-eyed *ebony* males in Fig. 3, S3 (boxing 8.39, wing threat 12.10, lunging 0.18, chasing 1.56), altogether suggesting that visual impairment alone does not cause increased aggression. p-values were obtained by Mann-Whitney U test (A-D); averages are shown with error bars representing SEM. (See Table S1 for n and statistical analysis for each experiment; n.s.=p>0.05, ****=p<0.0001.)

**Figure S6. In inter-genotype fights, *ebony* mutants show similar aggressive behaviors as controls.** (A) *ebony* mutants (green), compared to controls (gray), exhibited similar levels of total aggression in both intra-genotype fights (p=0.3327) and inter-genotype fights (p=0.6825) (n=number of videos). (B) Stacked bars display the behavioral indices for specific aggressive behaviors in intra-genotype fights featuring whole-body and glial-specific *ebony* knockdowns and genetic controls (n=number of videos; wing threat=light pink; chasing=pink; lunging=fuchsia; boxing=purple). (C-H) In inter-genotype fights, *ebony* mutants (green), compared to controls (gray), exhibited: similar (C) lunge counts per fly (p=0.3119); (D) latency to first lunge (p=0.4453); (E) boxing index (p=0.3984); (F) wing threat index (p=0.6604); (G) lunging index (p=0.9665); and (H) chasing index (p=0.0812); n=number of flies. (I) Bar graph showing the percentage of videos recorded in the more ecologically relevant arena that have fights demonstrating a dominance pattern. *ebony* intra-genotype fights (green) had a similar percentage of dominance-containing videos as control intra-genotype fights (gray) (p=0.6499); n=number of videos. p-values were obtained by Mann-Whitney U test (A, intra-genotype fights, C, E-F), unpaired Student’s t-test with Welch’s correction (A, inter-genotype fights, D, G-H), and Fisher’s exact test (I). Averages are shown with error bars representing SEM. (See Table S1 for n and statistical analysis for each experiment; n.s.=p>0.05.)
