# Supplementary material for: Drosophila mutants lacking the glial neurotransmitter-modifying enzyme Ebony exhibit low neurotransmitter levels and altered behavior
Source: Sci Rep. 2023 Jun 27;13:10411. doi: 10.1038/s41598-023-36558-7 (PMC10300103; doi:10.1038/s41598-023-36558-7)
Supplement: Supplementary file 8 — Supplementary Table S1. [file 41598_2023_36558_MOESM8_ESM.pdf]

Table S1: Here we provide sample sizes and statistical tests for data shown in each figure.

Figure 1 has no figures requiring statistical tests.

Sample sizes and statistical tests for data in Figure 2:

Graph	Sample size (N)	Stat test	P values	Justification for stat test
B, total courtship index	*CS* = 49<br>*ebony* = 45	Mann-Whitney U Test	<0.0001	Both data sets failed D'Agostino & Pearson and Shapiro-Wilk test
C, wing extension index	*CS* = 49<br>*ebony* = 45	Mann-Whitney U Test	<0.0001	Both data sets failed D'Agostino & Pearson and Shapiro-Wilk test
D, chasing index	*CS* = 49<br>*ebony* = 45	Mann-Whitney U Test	<0.0001	*CS* failed D'Agostino & Pearson and Shapiro-Wilk test
E, attempted mounting	*CS* = 49<br>*ebony* = 45	Mann-Whitney U Test	0.0082	Both data sets failed D'Agostino & Pearson and Shapiro-Wilk test
F, latency to court	*CS* = 49<br>*ebony* = 42	Mann-Whitney U Test	<0.0001	Both data sets failed D'Agostino & Pearson and Shapiro-Wilk test
G, total courtship index	*CS*, female target = 42<br>CS, male target = 38<br><br>*ebony*, female target = 40<br>*ebony*, male target = 42	Kruskal-Wallis Test (Dunn's post hoc test)	*CS*, female target vs. *CS*, male target: p=0.0211<br><br>*ebony*, female target vs. *ebony*, male target: p>0.9999<br><br>*CS*, female target vs. *ebony*, female target: p<0.0001<br><br>*CS*, male target vs. *ebony*, male target: p=0.2585	Both *ebony* courting a female target and *ebony* courting a male target failed the D' Agostino & Pearson test and Shapiro-Wilk test. *CS* courting a male target also failed Shapiro-Wilk test.

Sample sizes and statistical tests for data in Figure 3:

Graph	Sample size (N)	Stat test	P values	Justification for stat test
B, boxing index	*CS* = 30<br>*ebony* = 31	Mann-Whitney U Test	<0.0001	*CS* failed D'Agostino & Pearson and Shapiro-Wilk test
C, wing threat index	*CS* = 30<br>*ebony* = 31	Mann-Whitney U Test	<0.0001	Both failed D'Agostino & Pearson and *CS* failed the Shapiro-Wilk test
D, lunging index	*CS* = 30<br>*ebony* = 31	Mann-Whitney U Test	0.0497	Both passed D'Agostino & Pearson but both failed Shapiro-Wilk test
E, latency to fight	*CS* = 28<br>*ebony* = 30	Mann-Whitney U Test	0.0002	*CS* failed D'Agostino & Pearson and Shapiro-Wilk test
G, boxing in a more ecologically relevant arena	*CS* = 20<br>*ebony* = 12	Fisher's exact test	<0.0001	Fisher's exact test chosen for binary comparisons
H, lunging in a more ecologically relevant arena	*CS* = 10<br>*ebony* = 11	Mann-Whitney U Test	0.0025	Both data sets failed the Shapiro-Wilk test

Sample sizes and statistical tests for data in Figure 4:

Graph	Sample size (N)	Stat test	P values	Justification for stat test
D, average activity per hour	*CS* = 30<br>*ebony* = 21	Unpaired t test with Welch's correction	0.4372	All data sets passed normality test
E, sleep per 24 hr period	*CS* = 30<br>*ebony* = 21	Unpaired t test with Welch's correction	<0.0001	All data sets passed normality test
F, daytime bout number	*CS* = 30<br>*ebony* = 21	Unpaired t test with Welch's correction	0.0038	All data sets passed normality test

G, nighttime bout number	*CS* = 30<br>*ebony* = 21	Unpaired t test with Welch's correction	0.0001	All data sets passed normality test
H, bout length (day and night)	*CS* = 30<br>*ebony* = 21	Mann-Whitney U Test	0.0002	*CS* failed D'Agostino & Pearson and Shapiro-Wilk test
I, daytime bout length	*CS* = 30<br>*ebony* = 21	Mann-Whitney U Test	0.0006	Both data sets failed D'Agostino & Pearson and Shapiro-Wilk test
J, nighttime bout length	*CS* = 30<br>*ebony* = 21	Mann-Whitney U Test	0.0005	*CS* failed D'Agostino & Pearson and Shapiro-Wilk test

Sample sizes and statistical tests for data in Figure 5:

Graph	Sample size (N)	Stat test	P values	Justification for stat test
B, qPCR	*Gal4* control = 4<br>*UAS* control = 4<br>*tubulin>ebony RNAi* = 4	Ordinary one-way ANOVA (Dunnett's multiple comparisons test)	*Gal4* control vs. *tubulin>ebony RNAi*: p<0.0001<br><br>*tubulin>ebony RNAi* vs. *UAS* control: p<0.0001	All data passed Shapiro-Wilk test; N is too small for the other three tests.
C, boxing and wing threat index	*Gal4* control = 20<br>*UAS* control = 21<br>*tubulin>ebony RNAi* = 21	Kruskal-Wallis test ((Dunn's post hoc test)	*Gal4* control vs. *tubulin>ebony RNAi*: p=0.0225<br><br>*tubulin>ebony RNAi* vs. *UAS* control: p<0.0001	*Gal4* control and *UAS* control failed the D'Agostino & Pearson test and *UAS* control failed the Shapiro-Wilk test
D, total courtship	*Gal4* control = 44<br>*UAS* control = 36<br>*tubulin>ebony RNAi* = 33	Kruskal-Wallis test ((Dunn's post hoc test)	*Gal4* control vs. *tubulin>ebony RNAi*: p<0.0001<br><br>*tubulin>ebony RNAi* vs. *UAS* control: p<0.0001	*tubulin>ebony RNAi* failed both the D'Agostino & Pearson test and Shapiro-Wilk test

E, sleep per 24 hr period	*Gal4* control = 40<br>*UAS* control = 27<br>*tubulin>ebony RNAi* = 39	Ordinary one-way ANOVA (Dunnett's multiple comparisons test)	*Gal4* control vs. *tubulin>ebony RNAi*: p<0.0001<br><br>*tubulin>ebony RNAi* vs. *UAS* control: p<0.0001	All data sets passed normality test
F, boxing and wing threat index	*Gal4* control = 27<br>*UAS* control = 21<br>*repo>ebony RNAi* = 32	Kruskal-Wallis test ((Dunn's post hoc test)	*Gal4* control vs. *repo>ebony RNAi*: p<0.0001<br><br>*repo>ebony RNAi* vs. *UAS* control: p=0.0096	*repo>ebony RNAi* and *UAS* control failed D'Agostino & Pearson test and Shapiro-Wilk test
G, total courtship	*Gal4* control = 43<br>*UAS* control = 45<br>*repo>ebony RNAi* = 55	Kruskal-Wallis test ((Dunn's post hoc test)	*Gal4* control vs. *repo>ebony RNAi*: p<0.0001<br><br>*repo>ebony RNAi* vs. *UAS* control: p<0.0001	*repo>ebony RNAi* and *UAS* control failed D'Agostino & Pearson test and *repo>ebony RNAi* failed Shapiro-Wilk test
H, sleep per 24 hr period	*Gal4* control = 32<br>*UAS* control = 32<br>*repo >ebony RNAi* = 32	Ordinary one-way ANOVA (Dunnett's multiple comparisons test)	*Gal4* control vs. *repo>ebony RNAi*: p<0.0001<br><br>*repo>ebony RNAi* vs. *UAS* control: p=0.0099	All data sets passed the D'Agostino & Pearson test and Shapiro-Wilk test

Sample sizes and statistical tests for data in Figure 6:

Graph	Sample size (N)	Stat test	P values	Justification for stat test
A, NT quantification, histamine	*CS* = 4<br>*ebony* = 4	Unpaired t test with Welch's correction	0.0004	All data passed Shapiro-Wilk test; N is too small for the other three tests.
B, NT quantification, dopamine	*CS* = 4<br>*ebony* = 4	Unpaired t test with Welch's correction	0.0410	All data passed Shapiro-Wilk test; N is too small for the other three tests.
C, NT quantification, serotonin	*CS* = 4<br>*ebony* = 4	Unpaired t test with Welch's correction	0.0457	All data passed Shapiro-Wilk test; N is too small for the other three tests.
D, NT quantification, octopamine	*CS* = 4<br>*ebony* = 4	Unpaired t test with Welch's correction	0.1084	All data passed Shapiro-Wilk test; N is too small for the other three tests.

Sample sizes and statistical tests for data in Figure 7:

Graph	Sample size (N)	Stat test	P values	Justification for stat test
C, percent of fights won per video	*CS* vs. *CS* = 30<br>*ebony* vs. *ebony* = 31<br>*ebony* vs. *CS* = 31	Kruskal Wallis (Dunn's post hoc test)	All p values shown here are for Kruskal Wallis:<br><br>"*CS* vs. *CS*" vs. "*ebony* vs. *ebony*": p=0.0057<br><br>"*CS* vs. *CS*" vs. "*ebony* vs. *CS*": p>0.9999<br><br>"*ebony* vs. *ebony*" vs. "*ebony* vs. *CS*": p=0.0339	"*CS* vs *CS*" failed Shapiro-Wilk test
D, percent of fights leading to dominance per video	*CS* vs. *ebony* = 31<br>*ebony* vs. *CS* = 31	Mann Whitney-U test	0.0175	*CS* failed the D'Agostino & Pearson test and the Shapiro-Wilk test

Sample sizes and statistical tests for data in Figure S1:

Graph	Sample size (N)	Stat test	P values	Justification for stat test
B, qPCR	*CS* = 4<br>*ebony* = 4	Unpaired t test with Welch's correction	0.0001	All data passed Shapiro-Wilk test; N is too small for the other three tests.

Sample sizes and statistical tests for data in Figure S2:

Graph	Sample size (N)	Stat test	P values	Justification for stat test
B, total courtship index	*CS* = 17<br>*ebony* = 44	Mann-Whitney U Test	<0.0001	*ebony* failed D'Agostino & Pearson and Shapiro-Wilk test
C, courtship latency	*CS* = 17<br>*ebony* = 32	Mann-Whitney U Test	<0.0001	Both data sets failed D'Agostino & Pearson and Shapiro-Wilk test
D, copulation	*CS* = 54<br>*ebony* = 61	Fisher's exact test	<0.0001	Fisher's exact test chosen for binary comparisons

Sample sizes and statistical tests for data in Figure S3:

Graph	Sample size (N)	Stat test	P values	Justification for stat test
A, percent of videos with boxing	*CS* = 30<br>*ebony* = 31	Fisher's exact test	0.0159	Fisher's exact test chosen for binary comparisons
B, chasing index	*CS* = 30<br>*ebony* = 31	Mann-Whitney U Test	<0.0001	Both passed D'Agostino & Pearson but both failed Shapiro-Wilk test
C, latency to lunge in a more ecologically relevant arena	*CS* = 10<br>*ebony* = 12	Unpaired t test with Welch's correction	0.1962	All data sets passed normality test

Sample sizes and statistical tests for data in Figure S4:

Graph	Sample size (N)	Stat test	P values	Justification for stat test
Pysolo activity index	*CS* = 30<br>*ebony* = 21	Unpaired t test with Welch's correction	<0.0001	All data sets passed normality test

Sample sizes and statistical tests for data in Figure S5:

Graph	Sample size (N)	Stat test	P values	Justification for stat test
A, boxing index	*wCS* = 34<br>*w;;ebony* = 29	Mann-Whitney U Test	<0.0001	*wCS* failed both tests
B, wing threat index	*wCS* = 34<br>*w;;ebony* = 29	Mann-Whitney U Test	<0.0001	*w;;ebony* failed both tests
C, lunging index	*wCS* = 34<br>*w;;ebony* = 29	Mann-Whitney U Test	0.1586	*wCS* failed both tests, *w;;ebony* failed the Shapiro-Wilk test
D, chasing index	*wCS* = 34<br>*w;;ebony* = 29	Mann-Whitney U Test	<0.0001	*w;;ebony* failed the D'Agostino & Pearson test

Sample sizes and statistical tests for data in Figure S6:

Graph	Sample size (N)	Stat test	P values	Justification for stat test
A, total aggression	*CS* vs. *CS* = 30<br>*ebony* vs. *ebony* = 31<br><br>*CS* vs. *ebony* = 24<br>*ebony* vs. *CS* = 24	Mann Whitney-U test<br>& Unpaired T test with Welch's correction	For intra-genotype fights, p=0.3327<br><br>For inter-genotype fights, p=0.6825	For intra-genotype data set, "*CS* vs *CS*" failed Shapiro-Wilk test<br><br>For the inter-genotype data set, all data set passed normality test
C, lunge count per fly	*CS* vs. *ebony* = 25<br>*ebony* vs. *CS* = 25	Mann-Whitney U Test	0.3119	Both data sets failed D'Agostino & Pearson and Shapiro-Wilk test
D, latency to lunge	*CS* vs. *ebony* = 21<br>*ebony* vs. *CS* = 21	Unpaired T test with Welch's correction	0.4453	All data sets passed normality test
E, boxing index	*CS* vs. *ebony* = 24<br>*ebony* vs. *CS* = 24	Mann-Whitney U Test	0.3984	*CS* failed the D'Agostino & Pearson test and the Shapiro-Wilk test. *Ebony* failed the Shapiro-Wilk test
F, wing threat index	*CS* vs. *ebony* = 24<br>*ebony* vs. *CS* = 24	Mann-Whitney U Test	0.6604	*ebony* failed the D'Agostino & Pearson test and the Shapiro-Wilk test
G, lunging index	*CS* vs. *ebony* = 24<br>*ebony* vs. *CS* = 24	Unpaired T test with Welch's correction	0.9665	All data sets passed normality test
H, chasing index	*CS* vs. *ebony* = 24<br>*ebony* vs. *CS* = 24	Unpaired T test with Welch's correction	0.0812	All data sets passed normality test
I, fights leading to dominance in a more ecologically relevant arena	*CS* = 8<br>*ebony* = 11	Fisher's exact test	0.6499	Fisher's exact test chosen for binary comparisons
